# Monitoring the abundance of flying insects and atmospheric conditions during a 9-month campaign using an entomological optical sensor

**DOI:** 10.1038/s41598-023-42884-7

**Published:** 2023-09-20

**Authors:** Topu Saha, Adrien P. Genoud, Gregory M. Williams, Benjamin P. Thomas

**Affiliations:** 1 Department of Physics, New Jersey Institute of Technology, Newark, NJ USA; 2 Institute of Light and Matter, Claude Bernard University, Lyon, France; 3 Center for Vector Biology, Rutgers University, New Brunswick, NJ USA

**Keywords:** Optical sensors, Population dynamics, Entomology

## Abstract

Monitoring the dynamics of insect populations is key to assessing the impact of human activities on insect populations. However, traditional methodologies relying on physical traps have inherent limitations in accurately monitoring insect abundance. Here, we present findings from a 9-month campaign conducted in New Jersey, USA, utilizing a near-infrared optical sensor known as eBoss. From April to December 2022, the eBoss derived the aerial density (insect/m^3^) and biomass density (mg/m^3^) with a 1-min resolution from a total of 302,093 insect observations. The data collected were analyzed in relation to air temperature, relative humidity, and wind speed. The results revealed that the abundance of flying insects exhibited an initial increase from April to June, reaching a peak of 0.094 insect/m^3^ and 1.34 mg/m^3^, followed by a subsequent decline towards the end of the year. Our investigation showed a surge in insect abundance above 12.5 °C, with particularly high levels observed between 19 and 31 °C. The impact of relative humidity and wind speed on insect populations was also explored. Overall, this campaign demonstrated the efficacy of photonic sensors in gathering novel and extensive data for the field of entomology, paving the way for improved understanding and management of insect populations.

## Introduction

Insects are the most abundant and diverse animal on the planet, playing vital roles in ecosystems, agriculture, and impacting human society in many ways. They serve as pollinators, decomposers, predators, and contribute to the food chain of many animals, including humans. The decreasing abundance and diversity of insect species has become a significant worry among both the scientific community and the general public^1–3^. Although there is variability across species, location, and time, several studies indicate a decline in population abundance at a rate of approximately 1–2% per year^4–8^. The monitoring of insect population is a crucial aspect of entomology, but the current tools available to researchers are limited in their capability to accurately assess insect abundance. Researchers commonly use interception traps such as malaise traps or attractant traps that use bait like light, pheromones, food, or CO_2_^9–15^. While these methods can accurately identify the family, genus, species, and sex of captured insects through expert identification or DNA barcoding, they have some drawbacks. Traps still require time-consuming and expensive laboratory analysis where insects must be identified and weighed^16–18^. As a result, the price and the complexity of operating large networks of physical traps are great barriers in the effort to continuously monitor insect population over large spatial and temporal scales. Entomologists continue to lack large-scale and long-term data on abundance and biomass trends. Moreover, the destructive sampling process used in traps impedes any prospect of significantly scaling up their use. They can have negative impacts on potentially endangered species, in addition to skewing abundance measurements when a large number of insects are removed from the ecosystem. We believe that these limitations inherent to traps are significant drivers for the general lack of data on insect population dynamics, which is now considered a significant issue in the field of entomology^3,19,20^. This “data crisis”, as it is sometimes referred to, poses challenges in assessing ecosystem health, identifying potential threats to insect populations, and evaluating the effectiveness of implemented policies.

Over the last decade, there has been significant improvement in entomological optical systems and entomological lidars^21–37^. These advancements have the potential to complement trapping techniques and address some of the limitations associated with collecting abundance data. Specifically, optical systems can observe very large numbers of flying insects (over 1000/day) without interruption. In two previous studies^29,30^, we demonstrated that photonic sensors can estimate the aerial density (insect/m^3^) and the volumetric biomass density (mg/m^3^) for months with a temporal resolution in the minute range. While it is not the focus of this study, optical systems can also retrieve many morphological and optical properties of insects in their field of view, such as mass, wingbeat frequency and its harmonics, body and wing optical cross-sections, as well as polarimetric and spectroscopic properties to name a few. These parameters can be used to identify the species, sex group and even gravidity of specimen^25,26,30,38,39^ transiting through the field of view of the instrument, albeit with lower accuracy than physical traps.

The present study makes use of an optical sensor developed over the last three years at the New Jersey Institute of Technology. The sensor is referred to as the Entomological Bistatic Optical Sensor System, or *eBoss*, and is designed to monitor the aerial density of flying insects, as well as their volumetric mass density, simply referred to here as biomass density. This instrument is designed to be simple, affordable (≈ 2000 USD), automated and eye-safe. Therefore, it can be easily deployed in public settings. It has the ability to track both aerial and biomass densities with a 1 min-resolution and operate over long period of time with little to no human intervention. These features allow for short time scale studies of insect abundance, circadian rhythms, insect behaviors and the impacts of pesticide applications, to name a few. They also enable long-time scale studies of population dynamics, decline of insect abundance and the impacts of climate change or public policies.

As previously demonstrated, the eBoss can estimate the mass of individual insects and derives the biomass density within the volume of air probed by the instrument. The results of this previous study have been published in A. Genoud et al.^29^, where additional details about the experimental setup, data processing and analysis are provided. The system presented in this contribution has been deployed for nine consecutive months from April to December 2022, during which the evolution of the aerial density and biomass density are measured throughout the season and studied with respect to atmospheric conditions, such as temperature, relative humidity and wind speed. To our knowledge this is the first time that metrics related to insect abundance have been monitor with such high temporal resolution and over such long period of time, demonstrating that entomological photonic sensors may become a powerful tool to monitor insect populations.

## Methodology

### Experimental setup

**Figure 1:**
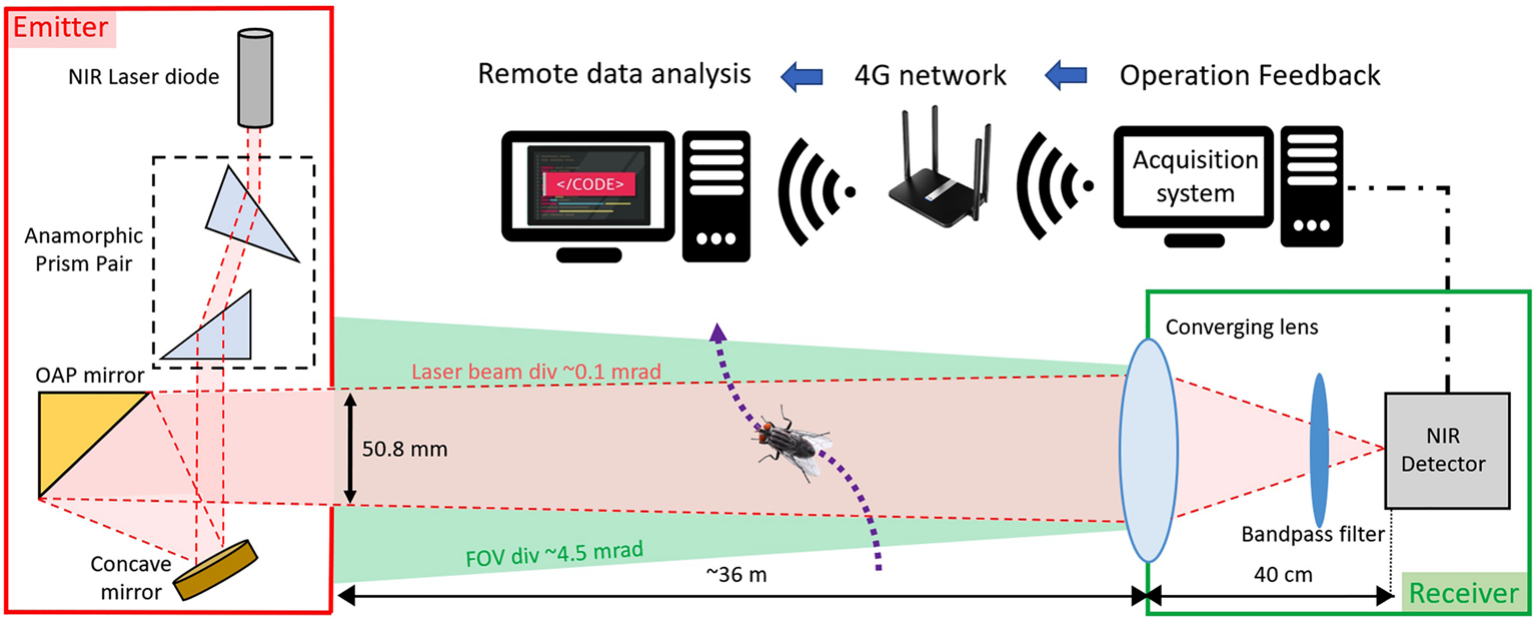
Optical layout of the eBoss deployed during the 2022 field campaign.

**Figure 2:**
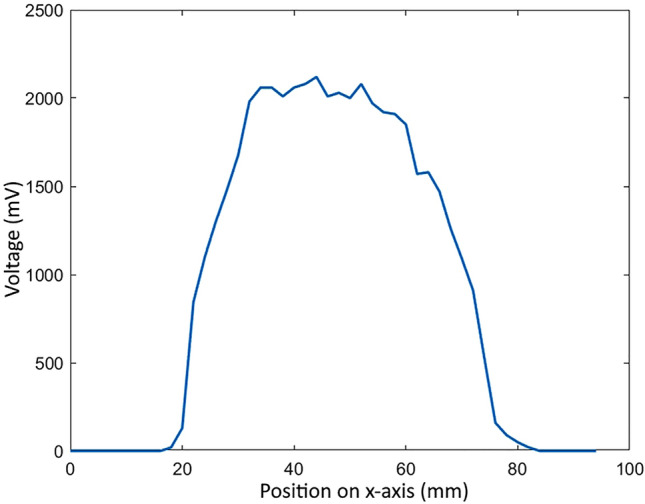
Spatial beam profile of the laser beam (x-axis).

This field experiment makes use of a novel eBoss instrument designed to be easily deployable in public settings. The eBoss optical layout is presented in Fig. 1. It consists of an emitter and a receiver sitting between 1 and 100 m from each other (36 m in this study). The laser source in the emitter is a 5mW continuous laser diode (CPS980, Thorlabs, USA). The laser wavelength is in the near-infrared spectral range, 980 nm at 20**°**C, and is outside the visible range of insects^40^. In addition, its intensity is too low to cause any significant heating, making the laser beam imperceptible to insects. Because the laser beam profile is initially elliptical, the beam is first transmitted through an anamorphic prism pair with 3× magnification to obtain a circular Gaussian beam. The beam is transmitted toward a concave mirror and is then reflected by an off-axis parabolic gold mirror (OAP mirror). Both mirrors act as a reflective beam expander, although only the center of the beam is reflected by the OAP mirror, so that the wings of the Gaussian beam are not reflected. As a result, the FWHM diameter of the beam reaches 50.8 mm and the energy density of the laser beam can be assumed to be homogenous (i.e. “flat top approximation”), this assumption is further discussed in Section “Data analysis”. The spatial profile of the beam taken at its center through its x-axis is displayed in Fig. 2. The beam is then directed horizontally over a distance of 36 m and propagates between 20 and 80 cm above the ground. The light is collected by the receiver at the end of the optical path via a large converging lens (ø 20 cm, f = 40 cm). The light goes through a spectral bandpass filter and is then focused onto the active area of a silicon amplified photodetector (PDA36A2, Thorlabs, USA). The spectral bandpass filter has a transmission above 95% from 950 to 1000 nm wavelength to minimize the contribution of the Sun and other unwanted light sources in the vicinity of the detector. The 50-nm wide spectral window is chosen to account for changes in laser wavelength due to large temperature fluctuations over the course of the season. The detector has an effective bandwidth of 90 kHz and has a large active area (3.6 × 3.6 mm). While it increases the field of view of the receiver, the large active area reduces the effect of the laser pointing jitter mainly caused by mechanical vibrations on the emitter side. The optical signal is recorded at a sampling frequency of 30,517 Hz using a 16-bit digitizer (M4i4420- × 8, Spectrum, USA) with a 5 V range. The acquisition system is integrated to a regular desktop computer. The system is protected from wind, rain and snow by a tent on both emitter and receiver sides. The desktop is connected via a 4G LTE router to transfer control data and monitor the proper functioning of the system. Additionally, a weather station (WS-1002-WIFI, Ambient Weather, USA) is positioned about 15 m away from the receiver to monitor the meteorological conditions such as temperature, rain, relative humidity, wind speed and direction as well as UV radiation level. The eBoss was installed in a field within the city of Secaucus (Hudson County, NJ, USA), it is approximately 40 × 10 m with tall grass bordered by a roughly 1 ha woodlot. The instrument was deployed on April 20th, and data was collected until December 21st, 2022.

During the previous 2021 campaign, the beam expansion was achieved using refractive lenses, as showed in A. Genoud et al.^29^. This new version of the eBoss uses a reflective beam expander which significantly reduces diffraction patterns observed over long distance (< 10 m) and provides a better beam quality than its previous version, specifically the energy density within the beam is more homogenous leading to better measurement of the optical cross section of insects and overall better signals.

### Data analysis

**Figure 3:**
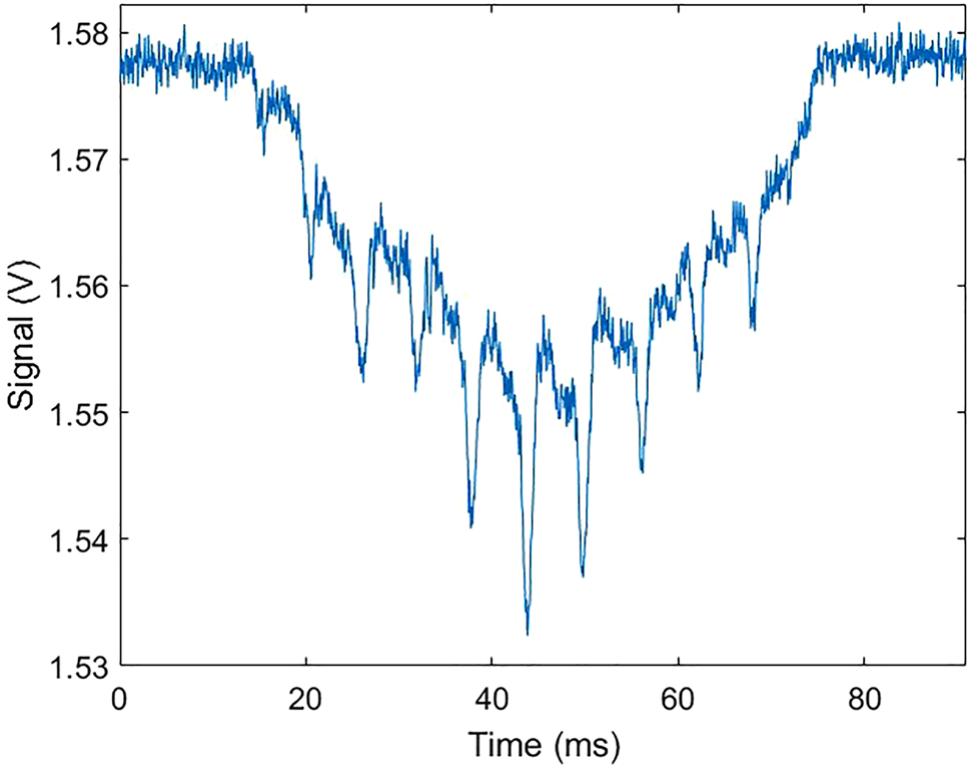
Typical signal recorded by the eBoss when an insect transits through the laser beam. The sharp drops in signal are caused by the rapid movement of the wings during the duration of the transit.

Figure 3 shows an example of a signal recorded by the eBoss when an insect flies through the probe volume of the system. The probe volume is defined as the intersection between the laser beam (red in Fig. 1) and the field of view of the detector (green in Fig. 1). In practice, the probe volume is identical to the volume covered by the laser beam. In the absence of any target in the probe volume, the recorded signal equals the voltage corresponding to the flux of photons received by the detector which defines the baseline signal. This baseline signal can change by a few percents over the course of a few hours due to variations in sunlight, change in optical extinction of the probed air or small drift in laser power. When an insect flies through the laser beam, the measured signal drops as part of the laser intensity is absorbed, diffracted, or scattered by the insect. Due to the movement of the wings as the insect transits through the beam, the optical attenuation cross section of the insect rapidly changes, creating sharp drops in signal which occurs every time the wings display a large apparent surface. These drops are visible in Fig. 3 where eleven wingbeat cycles occur during the transit.

When the insect transits through the probe volume (also referred to as an event), the signal displays a Gaussian-like envelope as the insect enters then exits the probe volume. The transit duration typically lasts around 100 ms, with events shorter than 10 ms being automatically discarded and the longest events approaching 1 s. Events can also be caused by non-insect targets, such as falling leaves, large pollens, water droplets or any other object entering the laser beam. In order to detect insect events, a detection algorithm is used to identify drops in signal below a certain threshold, and the wing patterns are used to discriminate between insects and other non-insect objects. This algorithm has been thoroughly described previously^29^. In the case of rain, water droplets create transit signals that can be distinguished from insect transit signals because of their absence of frequency components in the 10–900 Hz frequency range. Those events can be effectively discarded for light rain and do not prevent the proper collection of data. However, with heavy rain, there is a continuous flow of water droplets through the beam, making it impossible to identify potential transits.

### Aerial density

By measuring many events per day, the sensor can evaluate the aerial density of flying insects. Instead of simply counting the number of transits, this approach considers the time of transit of each event, related to the flight velocity of the insect. Fast flying insects, such as flies, are more likely to interact with the instrument as they cover more distance in a given time, but with a shorter time of transit. Slower insects such as butterflies or moths are less likely to enter the instrument’s field of view but will remain within the beam for a longer time. As a result, counting events inherently includes a bias toward fast flying insects, and is directly related to the probe volume of the instrument, making it a relative unit of abundance. The aerial density is calculated using Eq. (1), where the sum of all transit time is normalized by the duration of the measurement and the volume probed by the instrument, providing an absolute unit expressed in number of insects per meter cube of air. This approach has been discussed extensively in A. Genoud et al. and tested using a numerical simulation^24^.

1 $$\rho_{a} = \frac{{\mathop \sum \nolimits_{i} \Delta t_{i} }}{T \cdot V}$$

where ${\rho }_{a}$ is the aerial density expressed in insect/m^3^, ${\Delta t}_{i}$ is the transit time of event $i$, and $V$ is the probe volume of air. $T$ is the duration during which ${\rho }_{a}$ is measured and defines the temporal resolution of ${\rho }_{a}$.

### Biomass density

Because the cross section of the laser beam is known, the extinction cross section of the insect expressed in mm^2^ can be derived from the drop in voltage measured by the instrument. Assuming the energy density of the laser beam as constant within the probe volume, the insect attenuation cross section ${\sigma }_{i}$ can be derived by the following equation:

2 $$\sigma_{i} = \sigma_{L} \cdot \frac{{I_{0} - I_{T} }}{{I_{0} }}$$

where ${\sigma }_{L}$ is the cross section of the laser (≈ 2027 mm^2^), ${I}_{0}$ is the signal baseline value and ${I}_{T}$ is the minimum value of the transit signal. The cross section of the wings and the body can be differentiated by interpolating the local maximum during the transit signal, allowing for the retrieval of the attenuation cross-section of the wings and the optical cross section of the body separately (body here referring to all parts of the insects excluding the wings)^29^.

The mass ${m}_{i}$ of an insect $i$ expressed in mg can be retrieved by the following equation:

3 $$m_{i} = \eta \cdot \sigma_{i}^{3/2}$$

where $\eta$ is a factor allowing to convert the attenuation cross section of the insect into its mass. A previous laboratory study^29^ demonstrated this approach to be robust and the value for $\eta$ has been found to be equal to 0.157 mg/mm^3^ for the wet mass of the insect, and equal to 0.075 mg/mm^3^ for the dry mass of the insect. All biomass densities presented in the result section are based on the dry mass.

The volumetric biomass density of flying insects is then derived similarly to the aerial density by normalizing the sum of all mass by the time of observation and the total probe volume:

4 $$\rho_{b} = \frac{{\mathop \sum \nolimits_{i = 1}^{N} \frac{{\Delta t_{i} }}{T} \cdot m_{i} }}{V}$$

The time resolution of the aerial density ${\rho }_{a}$ or the biomass density ${\rho }_{b}$ is defined by $T$, which can be set to days, weeks or months to observe their long-term evolution or set to minutes or hours to observe shorter scale dynamics. The number of observed events, and therefore the retrieved aerial and biomass densities, is subject to stochastic fluctuations^24,25,30,41^. For this reason, there is a tradeoff between time resolution and uncertainty: high temporal resolution, in the hour or minute range, may present significant statistical fluctuations on the retrieved density while a longer time resolution, over days or weeks, may provide more robust results, but at the expanse of short time scale information.

To evaluate the biomass density, the assumption that the beam energy density is spatially homogenous (flat-top approximation) is used, however, as seen in Fig. 2, the beam spatial profile is not perfectly flat. Insects may transit through various levels of energy density, resulting in a potentially over or underestimated mass. In addition to this effect, some insects may not fully enter the beam and transit through its edge, resulting in an underestimation of their mass. Finally, the apparent surface of the insect may change depending on its orientation when inside the beam, which impacts the retrieved extinction cross section and therefore its retrieved mass. The overall error resulting from these effects was evaluated when the $\eta$ parameter was retrieved experimentally. The 95% confidence bounds for $\eta$ was (0.065, 0.085 mg/mm^3^), leading to an estimated relative error on the retrieved biomass density of ± 13.3%.

## Results

### Aerial and biomass densities

**Figure 4:**
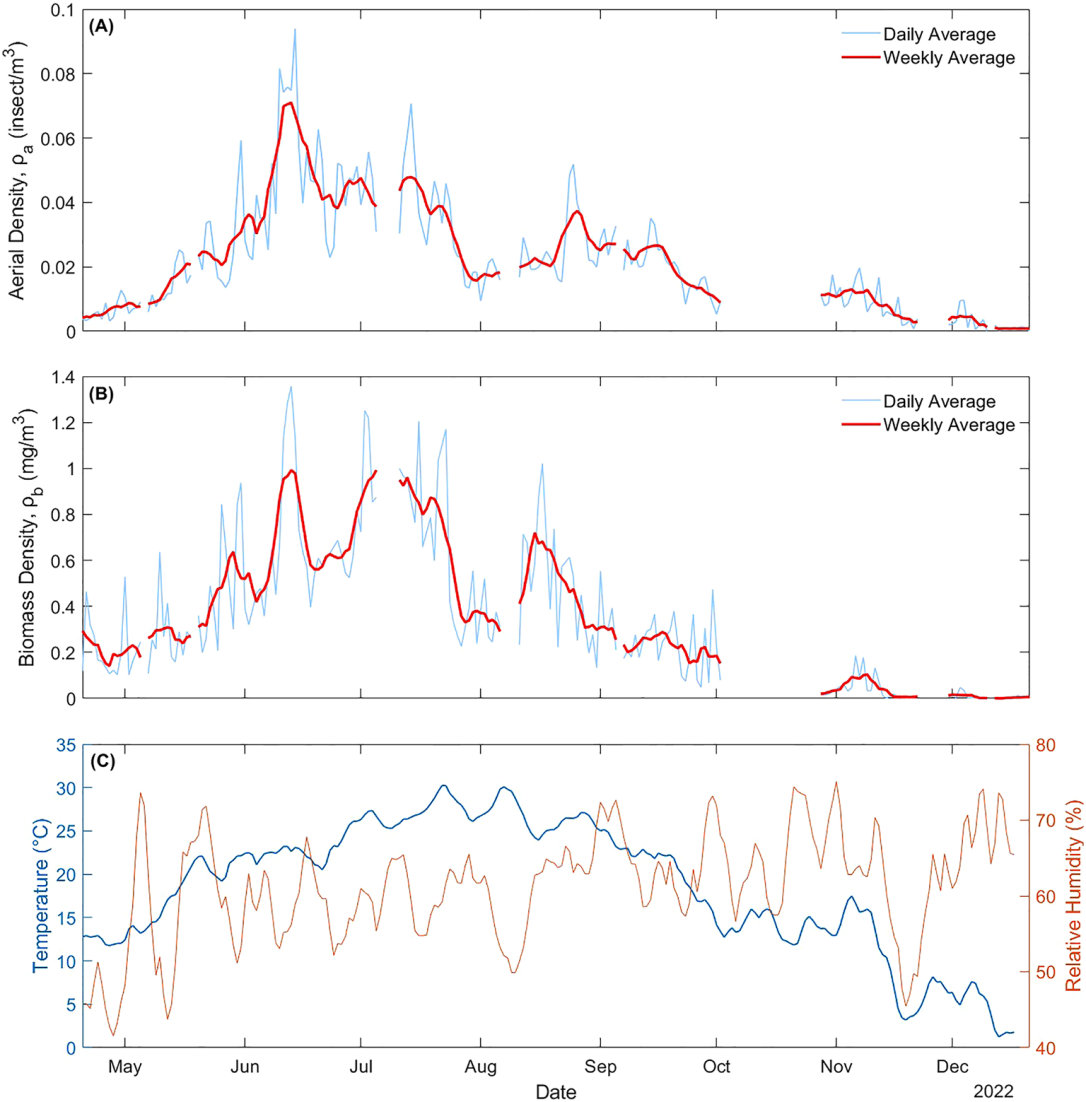
Aerial and biomass densities (**A** and **B**), and daily temperature and relative humidity (**C**).

Using the methodology described in Section “Data analysis”, both the aerial density and biomass density have been measured throughout the season and the results are displayed in Fig. 4. Both metrics are displayed using a daily average ($T = 24\;{\text{h}}$) along with the 7-day rolling average, the temperature and relative humidity. During the campaign, a total of 435,980 transits were recorded, of which 302,093 (69.3%) were identified as insect events. The data collection was interrupted multiple times during the campaign because of multiple power outages, acquisition software or computer malfunctions, and realignment and routine checkups of the system. In addition, data collected during intense rain or snow events were removed. When the duration of the interruption remained below 6 h per day, both daily ${\rho }_{a}$ and ${\rho }_{b}$ were calculated by subtracting its duration to $T$. When more than 6h of interruption occurs, the whole day was removed entirely as short periods of either high or low activity gain excessive weight within that day, leading to sometimes abnormally high or low densities. The resulting down time over total campaign time was 17.84%, mainly caused by one isolated malfunction of the instrument during the month of October (representing 55% of the total time down time).

Results show an increase in both biomass density and aerial density starting in April and reaching a maximum on June 13th and 14th, respectively. The aerial density reaches a maximum of 0.094 insect/m^3^ (or one insect for every 10.6 m^3^), while the biomass density reaches a maximum of 1.34 mg/m^3^. Past the peak in June, both densities slowly decrease until December, with a notable sharp decrease in August followed by a slight increase in September. Differences between the evolution of aerial and biomass densities indicate a change in the average mass of observed insects, potentially caused by new species emerging or disappearing.

**Figure 5:**
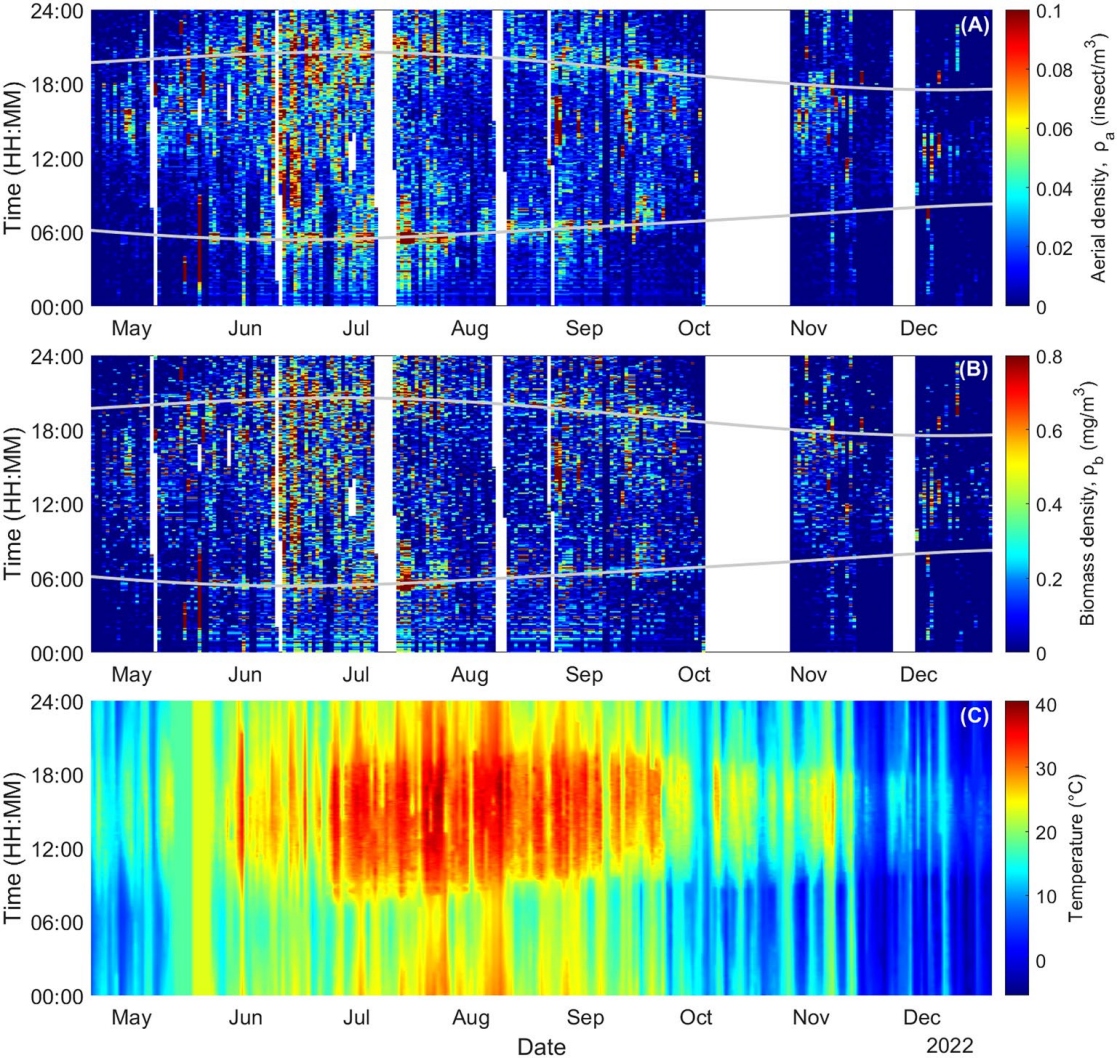
Aerial (**A**), biomass density (**B**) and temperature (**C**) as a function of date and time of day (EST, UTC-05:00) with a 1-min time resolution. White color indicates times when the eBoss was not operating, grey lines indicate the sunrise and sunset time.

The timestamp of each event allows for the monitoring of the aerial and biomass densities over a 24 h cycle with a time resolution in the minute range. Figure 5A and B show both ${\rho }_{a}$ and ${\rho }_{b}$ over the entire 9-month period and as a function of the time of day with a 1-min resolution. These figures highlight the ability of entomological photonic sensors to monitor insects’ activities over extended periods of time with little interruption and high temporal resolution. Figure 5C presents the color map of the temperature. The sunrise and sunset times are shown by a grey line. During the spring and fall months, both ${\rho }_{a}$ and ${\rho }_{b}$ display low values at night (< 0.01 insect/m^3^ and < 0.1 mg/m^3^). Warmer summer months show continuous activity at night, with maximal values around sunrise and sunset time.

### Impacts of atmospheric conditions

Insects’ activity is greatly influenced by the temperature of their bodies, which is almost entirely dependent on the atmospheric conditions^42–45^. For each insect event, both the temperature and relative humidity at the time of transit were recorded, allowing for the retrieval of the average aerial and biomass densities measured within a given temperature range and/or RH range. Equations (1) and (4) are used to evaluate the average aerial and biomass densities for a certain range of temperature and RH. In this case, the duration $T$ is equal to the total time of recording during which the temperature and RH were within the desired range. Therefore, while there is less data available during certain atmospheric conditions, the aerial and biomass densities are normalized over time. As a consequence, the occurrence of a given temperature or RH does not impact the retrieved aerial and biomass densities.

**Figure 6:**
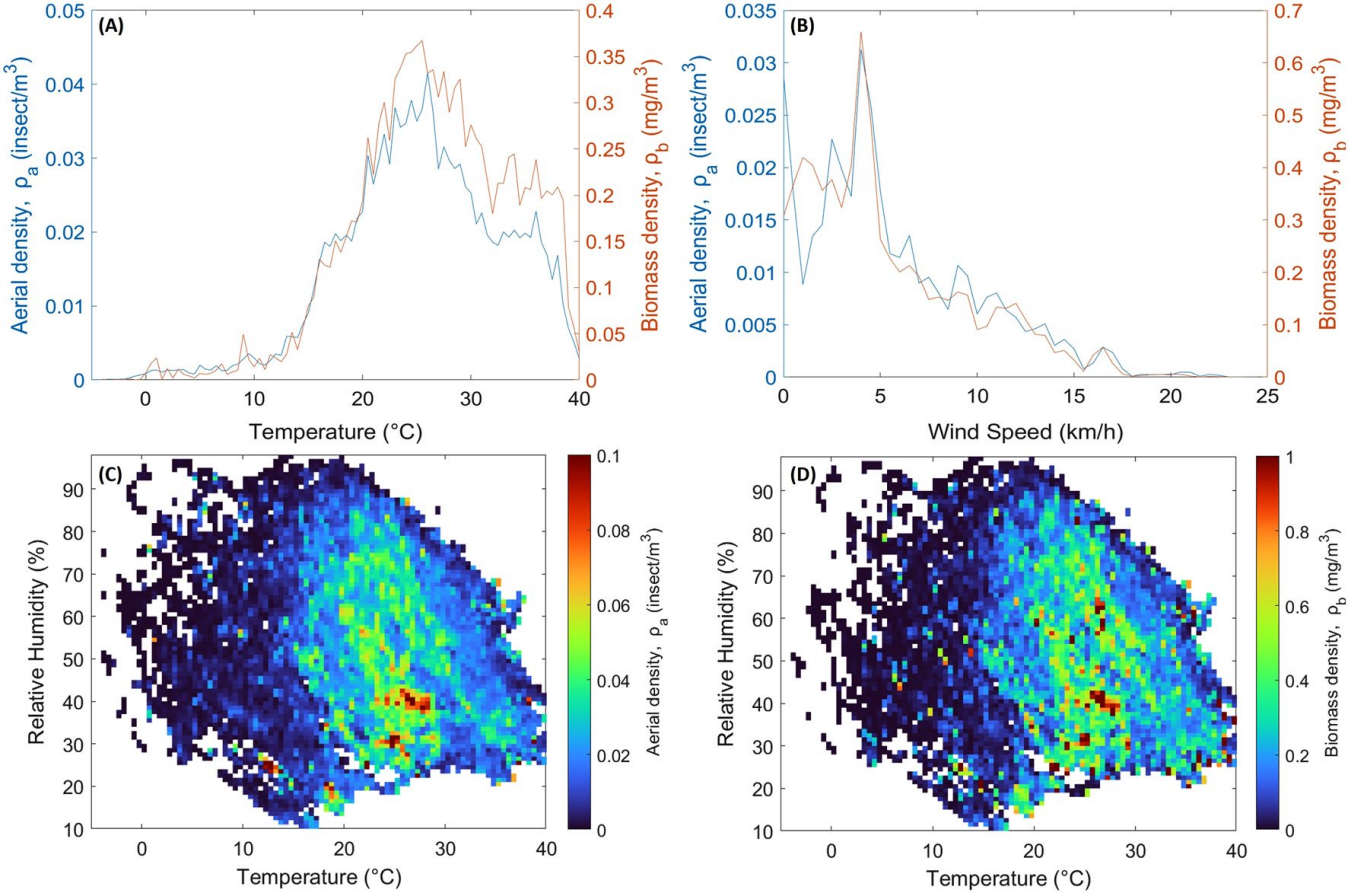
(**A**) Average aerial and biomass densities as a function of temperature (0.5 °C temperature interval). (**B**) Average aerial density as a function of wind speed (1 km\h interval). (**C** and **D**) Average aerial and biomass density as a function of temperature and relative humidity (0.5 °C and 1% intervals respectively), white color represents the absence of recorded data.

Figure 6A shows the average ${\rho }_{a}$ and ${\rho }_{b}$ by 0.5 °C temperature interval from the lowest to highest temperature recorded during the field experiment (− 5 to 40 °C). This figure shows that flying insects’ activity becomes much more pronounced when the temperature reaches 12.5 °C and above, with 95.7% of all activity recorded above this temperature. The highest activity was measured for temperatures between 19 and 31 °C, with a maximum at 26 °C. Both aerial and biomass densities sharply decrease when temperature exceeds 36.5 °C, which is agreement with previous observations^46^.

Similarly, the impact of wind speed on aerial and biomass densities was studied. However, the range of wind speed observed throughout the season is somewhat small, with the maximum wind speed measured reaching 23 km/h. Figure 6B presents the average densities ${\rho }_{a}$ and ${\rho }_{b}$ by wind speed interval (1 km/h). The aerial density appears to decrease with increasing wind speed, with no activity at all past 21 km/h, here as well in good agreement with A. P. Møller’s findings^47^.

The heat exchange between insects and their environment is a function of air temperature and the specific heat capacity of ambient air. The latter is greatly influenced by the relative humidity (RH). Figure 6C and D presents two colors plots, showing the average aerial density and biomass density found between a given temperature and RH range. As in Fig. 6A, the same favorable temperature range can be seen, although it appears that for high temperature (above 30 °C), insects are mostly active for low RH values. This would indicate that with high temperatures and high RH, insects are either less active or seek cooler environment due to excessive heat.

## Discussion and conclusion

In this study, the collection of weather-related data coupled with the high temporal resolution of the eBoss instrument enables the study of population dynamics as a function of atmospheric conditions. We observed that high abundance was measured mostly when temperatures were above 12.5 °C. The present study does not aim at providing an in-depth analysis of how atmospheric conditions may impact insect abundance, however, it demonstrates that optical sensors coupled with weather stations may be an effective way to collect data to better understand the complex relationships between weather/climate and insect population dynamics. With growing concerns that climate change may lead to significant shifts in insect populations, it appears necessary to develop such tools, and this study aims at demonstrating the potential of such an approach. This work highlights the effectiveness of entomological photonic sensors in continuously monitoring insect abundance over an entire season. The strengths of this approach, when compared to more common methods based on physical traps, are as follows:

- Firstly, we believe that this approach requires less supervision and personnel than physical traps, both in operating the instrument and analyzing its data. This can significantly reduce the cost associated with monitoring insect populations and opens the possibility of deploying large networks of sensors, allowing for data collection on insect populations at large temporal and spatial scales.
- Secondly, the recorded data benefits from a much higher temporal resolution compared to traps. Data with a 1-min resolution allows for the study of insect behavior shortly before, during, and after various events, such as rain or pesticide applications. It also provides a more accurate reading of conditions at the exact time an insect is observed, as demonstrated in the present study with temperature, relative humidity, and wind speed that are measured at the exact time when an insect is observed. Additionally, the eBoss can be used to study the short-term effects of various parameters that can be controlled by an operator to study insect behavior. For example, it could measure the impacts of light sources with various intensities or wavelengths, or the effects of chemicals released in the environment.
- Finally, optical sensors offer several benefits, as partially discussed in the introduction, including the ability to observe a high number of insects, non-destructive operation, minimal downtime, and providing measurements in absolute units such as insects/m^3^ and mg/m^3^.
- On the downside, this approach has the following limitations:
- Identifying insects down to the species level remains challenging. While some species and sex groups may be identified based on their wingbeat frequency and optical properties, certain species may remain indistinguishable from others. However, it is worth noting that the issue of identification using optical sensors has made rapid progress in recent years^25,26,30–33,38^, and it is a relatively new and currently active topic of research.
- The biomass density retrieved through this methodology is based on an estimate of the optical extinction cross section of the insects. Many factors other than the actual mass of the insect influence the retrieved cross section, such as the beam energy density, the orientation of the insects and its trajectory within the beam, as discussed in Section “Data analysis”. As a result, the mass estimate of one insect may potentially be under or overestimated. However, the biomass density is based on the sum of the retrieved mass from multiple transit signals. The number of transit signals considered to evaluate the biomass density depends on its temporal resolution. Assuming those effects are random, this statistically reduces their effect and should converge toward a more accurate value as the temporal resolution goes down.
- Any metrics related to abundance, such as aerial or biomass densities, are derived from measurements made within the probe volume of air and are therefore subject to statistical fluctuations. For instance, a significant increase in aerial density can be caused by a few insects randomly hovering near the laser beam, leading to high aerial density readings that may not be representative of the surrounding environment. In general, due to the highly heterogeneous spatial distribution of insects, moving the instrument a few meters may result in potentially significant differences in measured abundance. This issue is also present with physical traps; however, it is widely accepted that traps provide relative measurements of abundance (number of captured insects) for which the spatial heterogeneity is less of an issue, while optical sensors provide absolute measurements (insects/m^3^) and is therefore something to take into consideration.

## Data Availability

The datasets generated and analyzed during the current study are available from the corresponding author upon reasonable request.
